# TGF-β Signaling and Resistance to Cancer Therapy

**DOI:** 10.3389/fcell.2021.786728

**Published:** 2021-11-30

**Authors:** Maoduo Zhang, Ying Yi Zhang, Yongze Chen, Jia Wang, Qiang Wang, Hezhe Lu

**Affiliations:** ^1^ State Key Laboratory of Membrane Biology, Institute of Zoology, Chinese Academy of Sciences, Beijing, China; ^2^ Institute for Stem Cell and Regeneration, Chinese Academy of Sciences, Beijing, China; ^3^ University of Chinese Academy of Sciences, Beijing, China; ^4^ Centre for Systems Biology, Lunenfeld-Tanenbaum Research Institute, Mount Sinai Hospital, Toronto, ON, Canada; ^5^ College of Biological Sciences, China Agricultural University, Beijing, China

**Keywords:** TGF-β pathway, TGF-β, chemotherapy resistance, targeted therapy resistance, immunotherapy resistance

## Abstract

The transforming growth factor β (TGF-β) pathway, which is well studied for its ability to inhibit cell proliferation in early stages of tumorigenesis while promoting epithelial-mesenchymal transition and invasion in advanced cancer, is considered to act as a double-edged sword in cancer. Multiple inhibitors have been developed to target TGF-β signaling, but results from clinical trials were inconsistent, suggesting that the functions of TGF-β in human cancers are not yet fully explored. Multiple drug resistance is a major challenge in cancer therapy; emerging evidence indicates that TGF-β signaling may be a key factor in cancer resistance to chemotherapy, targeted therapy and immunotherapy. Finally, combining anti-TGF-β therapy with other cancer therapy is an attractive venue to be explored for the treatment of therapy-resistant cancer.

## Introduction

### Relationship Between TGF-β Signaling and Cancer Therapy Resistance

Cancer is a leading cause of death globally and there has been on-going efforts to find cures for it. In addition to surgical removal of tumors as well as radiotherapy, a plethora of chemical compounds and/or biological agents have been employed for the treatment of cancer. Chemotherapy, consisting of cytotoxic agents that aim to target highly proliferative cancer cells, was first introduced in the 1940’s (Goodman and Wintrobe, 1946; Farber et al., 1948; Falzone et al., 2018). Since then, chemotherapeutic drugs such as paclitaxel, cisplatin, and doxorubicin have become first-line treatments for a variety of cancers (Falzone et al., 2018). However, chemotherapy acts not only on tumor cells but also on normal cells, which often leads to severe side effects. In search for anti-tumor drugs with higher selectivity for tumor cells and fewer adverse effects towards normal cells, scientists designed inhibitors against key molecular targets involved in driving cancer progression; such therapeutic strategies belong to the category of targeted therapy. For example, kinase inhibitors against the epidermal growth factor receptor (EGFR), like gefitinib and erlotinib, are used for the treatment of non-small cell lung cancer (NSCLC) patients with activating mutations in the EGFR gene (Antonicelli et al., 2013).

In the past 10 years, a new class of anti-cancer therapy has emerged with great promise in inducing prolonged responses in cancer patients with advanced or metastatic cancers (Sharma et al., 2017). Using biological agents such as monoclonal antibodies against immune checkpoints, as well as genetically engineered T cells, cancer immunotherapy harnesses the patient’s immune system to recognize and eradicate tumors. In addition, researchers have been testing different combinations of cancer therapies to optimize therapeutic efficacy while minimizing unwanted side effects. Despite advancements in anti-cancer therapies, achieving relapse-free survival remains challenging, due to the emergence of primary or acquired resistance in response to treatment (Oppermann et al., 2016). In some cases, patients fail to respond to cancer treatment in the first place, suggesting that primary resistance, which often arises from pre-existing genetic mutations or epigenetic alterations in the tumor, is impeding therapeutic response. In other cases, patients respond initially to drug treatment but its efficacy diminishes over-time, which indicates the development of acquired resistance. In this scenario, recurrent tumors are often more aggressive and resistant to treatments. Like primary resistance, acquired resistance can be attributed to a number of factors including genetic mutations that allow tumors to evade attacks by cancer therapy and/or to activate alternative survival pathways. Drug resistance is associated with increased expression of drug efflux transporters, activated proliferation and anti-apoptotic signaling, enhanced cancer stemness, as well as evasion of immunosurveillance (Nussinov et al., 2017). A number of recent studies have shown that activation of transforming growth factor β (TGF-β) signaling was associated with drug resistance in a variety of cancers including melanoma (Sun et al., 2014), NSCLC (Soucheray et al., 2015), breast cancer (Palomeras et al., 2019), hepatocellular carcinoma (HCC) (Bhagyaraj et al., 2019), colorectal cancer (CRC) (Quan et al., 2019), squamous cell carcinoma (SCC) (Brown et al., 2017), osteosarcoma (OS) (Wang et al., 2019), prostate cancer (Song et al., 2018a), as well as in tumor-initiating cells of a few types of cancer (Yu et al., 2018; Batlle and Massagué, 2019; Tang et al., 2020; Taniguchi et al., 2020). Moreover, high levels of TGF-β in patients with breast cancer, NSCLC, HCC, CRC predicted a poor prognosis (Calon et al., 2015; Okada et al., 2018; Zhuang and Wang, 2018; Tauriello, 2019; Guo et al., 2020). As a result, extensive research has been conducted to explore the potential role of TGF-β signaling inhibitors as means to overcome cancer treatment resistance (Huang et al., 2012; Sun et al., 2014; Jenkins et al., 2015; Koetz-Ploch et al., 2017; Li et al., 2019a; Wang et al., 2019).

**FIGURE 1:**
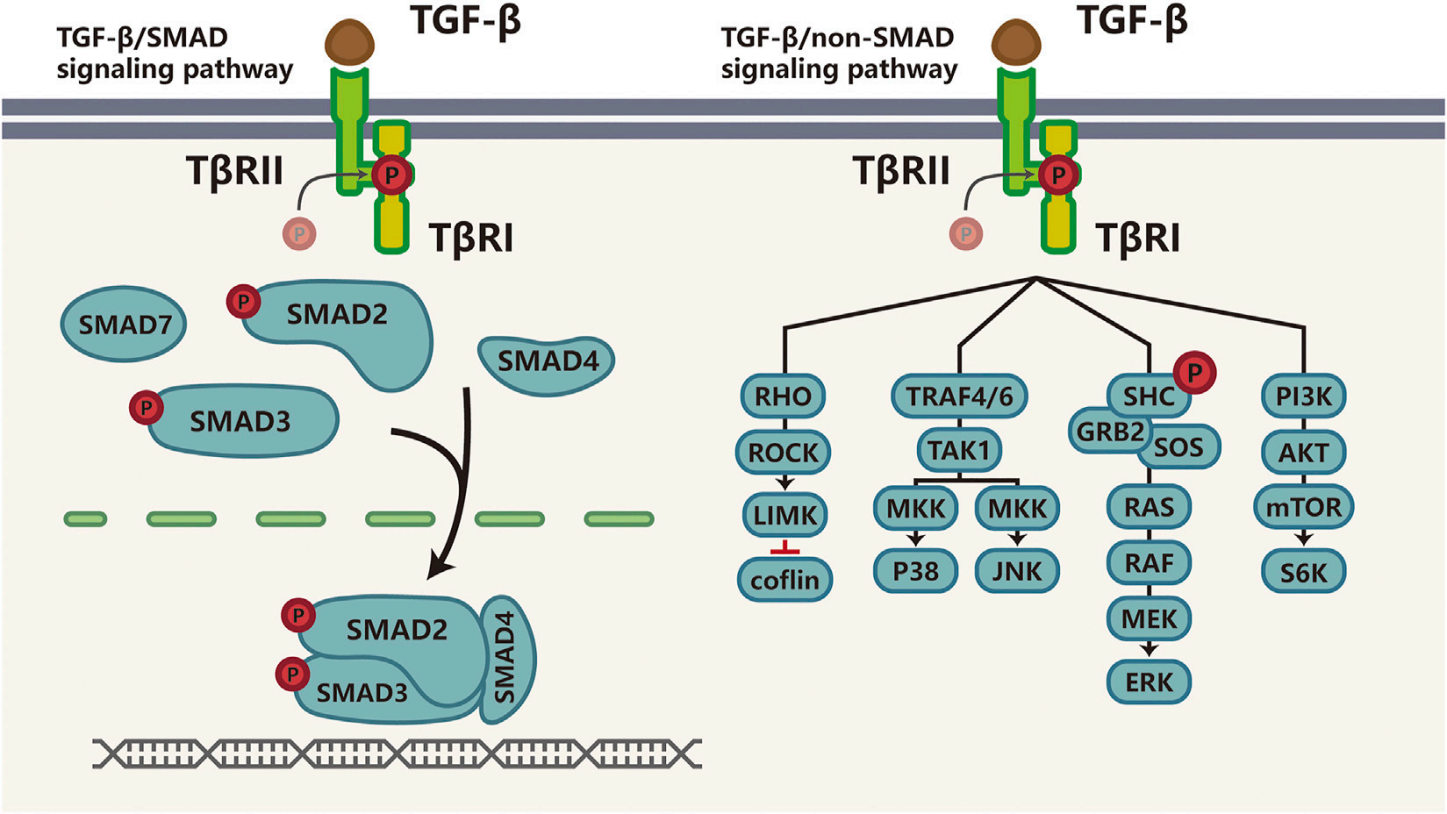
TGF-β signaling pathway TGF-β transduces signaling through SMAD or non-SMAD signaling pathways. Actived TGF-β binds to TGF-β ligand, Once TGF-β binds to TβRII, TβRI is recruited, phosphorylated and activated to phosphorylate the downstream mediators-SMAD2 and SMAD3; then SMAD4 binds to activated SMAD2 and SMAD3 to form heterotrimeric transcriptional complexes that translocate and relay this signaling into the nucleus to further regulate transcription. This is called canonical TGF-β/SMAD signaling pathway (right). The non-SMAD-dependent activation of the TGF-β pathway involves signaling via RHO GTPases, P38, JNK, ERK or MEKK, and PI3K-AKT (left). Abbreviations: P, phosphorylation; TβR, transforming growth factor (TGF)-β receptor; ROCK, RHO-associated coiled-coil containing protein kinase; LIMK, LIM kinase; TRAF, TNF receptor-associated factor; TAK1, TGF-β-activated kinase-1. JNK, c-Jun N-terminal kinase; SHC, SRC homology 2 domain-containing transforming protein; GRB2, growth factor receptor-bound protein 2; SOS, son of sevenless; MEK, mitogen-activated protein kinase; ERK, extracellular signal-regulated kinase; PI3K, phosphatidylinositol-4,5-bisphosphate; mTOR, mechanistic target of rapamycin.

The TGF-β superfamily, which comprises TGF-βs, Activins (Acts), Nodal, bone morphogenetic proteins (BMPs), growth and differentiation factors (GDFs) and anti-Müllerian hormone (AMH), is implicated in embryonic development, cell proliferation, differentiation, apoptosis, and immune responses (Yang et al., 2010; Moses et al., 2016). Importantly, TGF-β is a regulator of tissue homeostasis and cancer may result from dysregulated TGF-β signaling. For instance, during the embryo implantation period, TGF-β signaling is active in the endometrium to balance apoptosis and proliferation of endometrial cells (Dimitriadis et al., 2005; Latifi et al., 2019). In stratified rectal and genital epithelia lacking type II TGF-β receptor (TβRII) expression, TGF-β signaling is disrupted, leading to destabilized tissue homeostasis and the development of spontaneous SCCs in stratified epithelia (Guasch et al., 2007). There are three TGF-β receptor ligands: TGF-β1, TGF-β2, and TGF-β3. TGF-β signaling is activated when activated TGF-β ligands bind to TβRII to recruit type I TGF-β receptor (TβRI), leading to phosphorylation and activation of TβRI, which phosphorylates downstream mediators SMAD2 and SMAD3. SMAD4 then binds to SMAD2 and SMAD3 to form heterotrimeric complexes that translocate to the nucleus to regulate the transcription of target genes (Derynck and Zhang, 2003; Yang et al., 2010). Furthermore, SMAD6 and SMAD7 are part of a negative feedback loop that regulates the TGF-β pathway. The versatility in TGF-β receptor-ligand interaction is thoroughly discussed in a number of reviews (Derynck and Zhang, 2003; Zhang et al., 1007). In addition to the canonical signaling pathway described above, there are several SMAD-independent TGF-β pathways, which consist of RHO GTPases, P38, jun N-terminal kinase (JNK), mitogen-activated protein kinase (ERK or MKK), and phosphoinositide 3-kinase (PI3K)-AKT (Lee et al., 2007; Sorrentino et al., 2008; Heldin and Moustakas, 2016; Principe et al., 2017), as shown in Figure 1.

The functions of TGF-β are cell type- and context-dependent. Increasing evidence suggests that TGF-β signaling acts like a double-edged sword in tumor progression (Bierie and Moses, 2006; Massagué, 2008). In healthy cells and early-stage cancerous cells, activation of TGF-β signaling pathway promotes cell-cycle arrest and apoptosis; while in late-stage cancers, TGF-β signaling acts as an oncogene to induce metastasis and drug resistance (Bardeesy et al., 2006; Morikawa et al., 2016). For example, SMAD4 is phosphorylated by anaplastic lymphoma kinase (ALK) at Tyr95 in ALK-positive gastrointestinal, pancreatic and lung tumors, resulting in the inhibition of tumor suppressor activity of TGF-β (Zhang et al., 2019a). SMAD4 deletion accelerates the transformation from premalignant to malignant phenotype in pancreatic progenitors harboring Kirsten rat sarcoma virus (KRAS) mutations (Bardeesy et al., 2006; Zhang et al., 2019a). On the other hand, in advanced pancreatic ductal adenocarcinomas (PDAC), intact TGF-β/SMAD4 pathway facilitates cancer progression; in advanced prostate cancer, bone-borne TGF-β induces osteoclastogenesis and bone metastasis by activating chemokine (C-X-C motif) receptor 4 (CXCR4) (Bardeesy et al., 2006; Zhang et al., 2021). These studies provided concrete evidence for the tumor suppressive role of the TGF-β pathway in pre-malignant cells and oncogenic role in advanced cancers. In the past few decades, the dual role of TGF-β in tumorigenesis and tumor-suppression have been extensively studied (Roberts and Wakefield, 2003; Levy and Hill, 2006; Massagué, 2008) and a growing body of literature elucidated that TGF-β/SMAD pathway was activated in multi-therapy resistance. However, the mechanisms underlying TGF-β mediated-drug resistance are still being explored and existing evidence lacks consistency. In this review, we mainly focus on the role of TGF-β signaling in drug resistance. Here, we provide an overview of pre-clinical and clinical studies of TGF-β signaling in regulating cancer drug resistance, and offer our perspective on potential strategies to target TGF-β-mediated drug resistance in cancer patients.

## TGF-β Signaling and Resistance to Targeted Therapy

**FIGURE 2:**
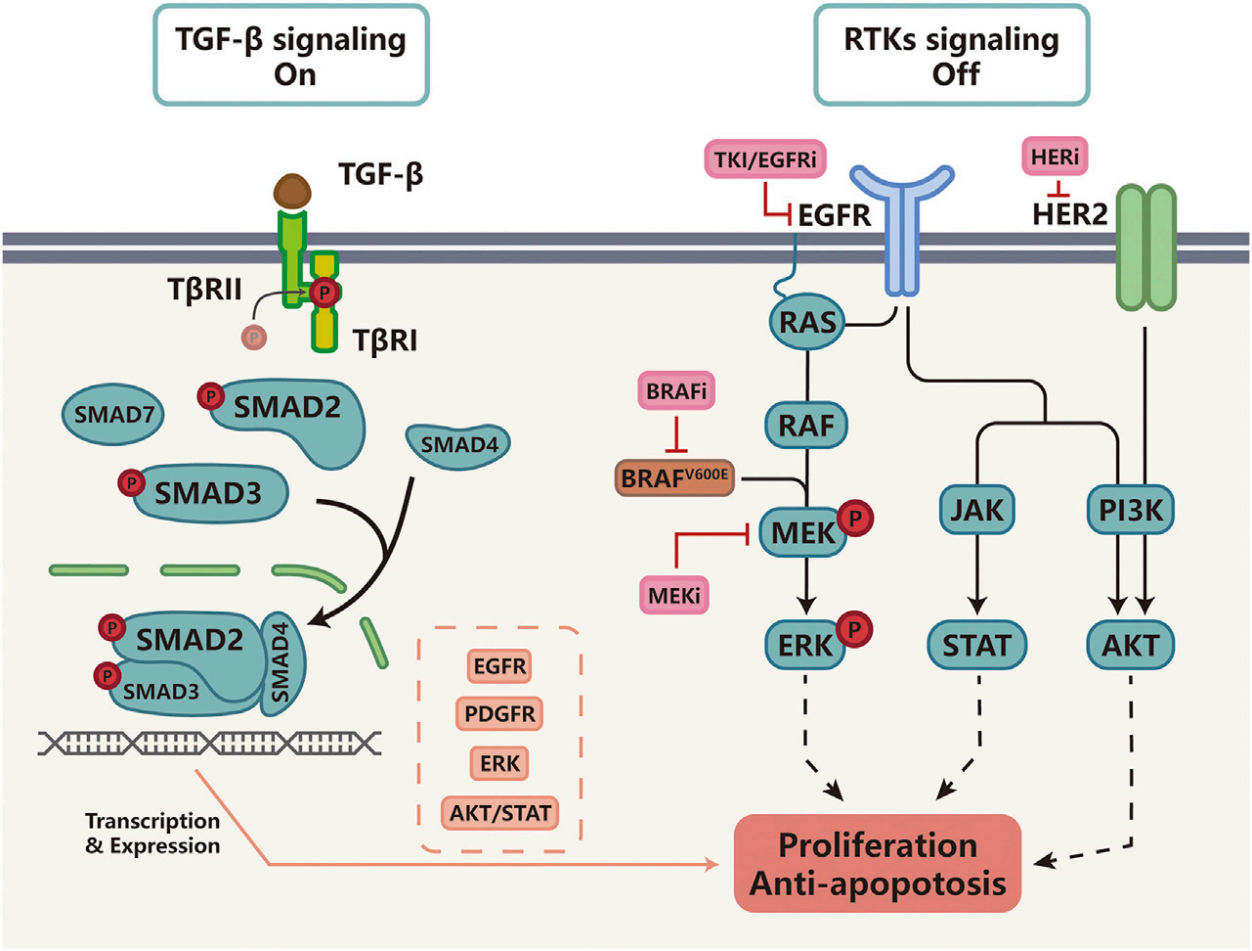
TGF-β signaling and resistance to targeted therapy Cancers with activating BRAF-mutations or EGFR-mutations as well as HER2-positive cancer are often treated with small molecular inhibitors against these molecular targets. For example, BRAF^V600E^ is often targeted by BRAFi such as vemurafenib, MEK by MEKi such as tramelinib, and HER2 by trastuzumab, Upon kinase inhibitor treatment, receptor tyrosine kinase (RTK) signaling is turned off. In cells that activate TGF-β-induced drug resistance, TGF-β signaling functions by increasing the expression of EGFR, PDGFR, ERK, AKT/STAT to activate alternative survival pathways and suppress apoptosis, protecting tumor cells from targeted therapy.

Targeted therapy acts by interfering with oncogenic cellular processes to selectively eradicate cancer cells, mainly including specific enzymes, growth factor receptors, and signal transducers. The first effective example of targeted therapy is the inhibition of the BCR-ABL1 oncogene in chronic myeloid leukemia (CML) (Salesse and Verfaillie, 2002). Subsequently, EGFR inhibitors (EGFRi) such as cetuximab, erlotinib and gefitinib were developed to treat EGFR-mutant NSCLC (Kazandjian et al., 2016); BRAF/MEK inhibitors (BRAFi/MEKi) were developed for BRAF-mutant melanoma; and epidermal growth factor receptor 2 inhibitors (HER2i) were developed for the treatment of HER2 positive (HER2^+^) breast cancer (Harbeck and Gnant, 2017). However, the efficacy of targeted therapy is often compromised by drug resistance and studies found that up-regulation of TGF-β signaling was a major driver of targeted therapy resistance (Brunen et al., 2013). Next, we summarize recent findings describing how TGF-β signaling helps tumor cells bypass pathway inhibition by activating alternative survival pathways or anti-apoptotic signaling pathways (Figure 2).

One example of TGF-β signaling-mediated resistance to targeted therapy was reported in cancer treated with BRAFi/MEKi (Sun et al., 2014; Lu et al., 2017; Bugide et al., 2020). The MAPK signaling pathway consists of kinases RAS, RAF, MEK, and ERK, which are essential for cell proliferation and survival. Hyper-activation of MAPK signaling occurs frequently in human cancers, such as melanoma, colorectal cancer, thyroid carcinoma, and hepatic cancer (Fang and Richardson, 2005; Santarpia et al., 2012; Lee et al., 2020). Treatment with BRAFi/MEKi, such as vemurafenib, sorafenib and trametinib, often results in remarkable disease regression initially, followed by the development of BRAFi/MEKi resistance (Rizos et al., 2014; Sun et al., 2014; Lu et al., 2017). Studies found that TGF-β signaling was frequently up-regulated in BRAFi-treated cancer cells (Faião-Flores et al., 2017; Bugide et al., 2020). Screening with a short hairpin RNA (shRNA) library focusing on chromatin regulators, Sun and his colleagues (Sun et al., 2014) discovered that TGF-β signaling was activated by the suppression of SRY-box transcription factor 10 (SOX10), thereby causing an up-regulation of EGFR and platelet-derived growth factor receptor-β (PDGFRB) signaling to confer resistance to MAPK inhibitors. In addition, TGF-β signaling was reported to mediate the up-regulation of microRNA-125a (miR-125a) expression and suppression of pro-apoptotic pathway, which accounted for the acquisition of BRAFi resistance in BRAF-mutant melanoma patients (Koetz-Ploch et al., 2017). Prete and others (Prete et al., 2018) demonstrated that in cancer cells with BRAF mutations, therapeutic escape from BRAFi/MEKi was facilitated by pericytes that secreted thrombospondin-1 (TSP-1) and TGF-β1, both of which led to a rebound of pERK1/2, pAKT and pSMAD3 (Fedorenko et al., 2015).

In addition to cancers with BRAF mutations, TGF-β signaling is also associated with therapy resistance in cancers with hyperactive EGFR. EGFR mutation or amplification are frequently detected in lung cancer; and studies suggest that activation of TGF-β pathway is associated with EGFRi/EGFR tyrosine kinase inhibitor (TKI)/cetuximab resistance (Yao et al., 2010; Bedi et al., 2012; Kurimoto et al., 2016; Li et al., 2016; Du et al., 2020; Kuo et al., 2020; Qiu et al., 2020). Approximately 30% of NSCLC patients with EGFR-mutations have no response to TKIs; such primary resistance can be attributed to mutations in the transforming growth factor beta receptor 1 (TGFBR1) gene and the resulting activation of TGF-β/SMAD signaling pathway-mediated mesenchymal-epithelial transition (EMT) (Zhong et al., 2017; Zhang et al., 2019b). Suppression of TGF-β signaling and down-regulation of Slug expression enhanced the gefitinib-sensitivity in TKI-resistant lung cancer cells (Qiu et al., 2020). Mechanistically, in EGFRi-resistant cancer cells, TGF-β signaling can be regulated by the binding of transcriptional factors to the promoter of TGFBR, or directly to the receptor itself. For example, zinc finger protein 32 (ZNF32) binds to the TβRII promoter to promote the expression of TβRII, while mediator complex subunit 12 (MED12) negatively regulates TβRII through physical interaction in the cytoplasm. Elevated expression of ZNF32 or reduced expression of MED12 up-regulate TGF-β signaling, resulting in MEK/ERK pathway activation to promote EGFRi-resistance in lung cancer (Huang et al., 2012; Li et al., 2016). Yao et al (2010) showed that both tumor cell-autonomous mechanisms and changes in the tumor microenvironment (TME) could activate the TGF-β–SMAD/IL6 signaling axis to drive erlotinib resistance, as IL6-induced STAT3 expression protected tumor cells from apoptosis (Yao et al., 2010). Moreover, TGF-β could activate AKT in an EGFR-independent fashion to inhibit cell apoptosis in EGFR-mutant cancers when treated with cetuximab and TKIs (Bedi et al., 2012; Wang et al., 2019). Further studies demonstrated that TGF-β down-regulated the expression of the stemness factor SOX2 to promote TKI tolerance (Kuo et al., 2020). In addition to the regulation of downstream targets to drive drug resistance, TGF-β regulates the alternative splicing of TGF-β-activated kinase 1 (TAK1) transcript into two isoforms: the short isoform TAK1∆E12 supports TGF-β-induced EMT and nuclear factor kappa B (NF-κB) signaling to confer resistance to afatinib (EGFR inhibitor), whereas the full-length isoform promotes TGF-β-induced apoptosis. Selective blockade of the expression of the short isoform by blocking TGF-β-induced alternative splicing of TAK1 may be potential avenue to overcome TGF-β-induced drug resistance (Tripathi et al., 2019).

Another example of TGF-β signaling-mediated resistance was reported in HER2 targeted therapy for HER2-positive cancers. Overexpression of HER2 occurs in 20–25% of human breast cancers; it is also observed in other types of cancers such as advanced gastric or gastroesophageal junction cancer (Boku, 2014). Trastuzumab is a humanized monoclonal antibody targeting HER2. Although it was approved for the treatment of metastatic cancers, a large cohort of the patients eventually developed trastuzumab resistance (Esteva et al., 2002). Studies found that TGF-β signaling pathway was consistently overexpressed in trastuzumab-resistant breast cancer cells and gastric cancer cells (Bai et al., 2014; Zhou et al., 2018). Treatment with exogenous TGF-β conferred insensitivity to trastuzumab in HER2-positive breast cancer cell lines, through up-regulating the expression of EMT and cancer stem cell (CSC) markers (Chihara et al., 2017). Growth differentiation factor 15 (GDF15)-mediated activation of TGF-β receptor-Src-HER2 signaling was also identified as a mechanism of trastuzumab resistance (Joshi et al., 2011). Combined with the activation of Src-focal adhesion kinase (FAK), TGF-β integrated HER2 and integrin signaling to promote cell survival and invasion to escape trastuzumab-induced apoptosis (Wang et al., 2009).

Other targeted therapy with resistance mechanisms associated with TGF-β signaling include CD4/6 inhibitor (Palbociclib), FAK inhibitor (VS-4718), androgen receptor inhibitors (enzalutamide), and BET inhibitor (Liu and Korc, 2012; Lin et al., 2013; Shi et al., 2016; Liu et al., 2017; Song et al., 2018b; Cornell et al., 2019; Paller et al., 2019). Taken together, these studies clearly demonstrated that TGF-β signaling pathway had an essential role in the development of resistance to targeted therapy against a variety of oncogenic pathways across different malignancies.

## TGF-β Signaling and Resistance to Chemotherapy

**FIGURE 3:**
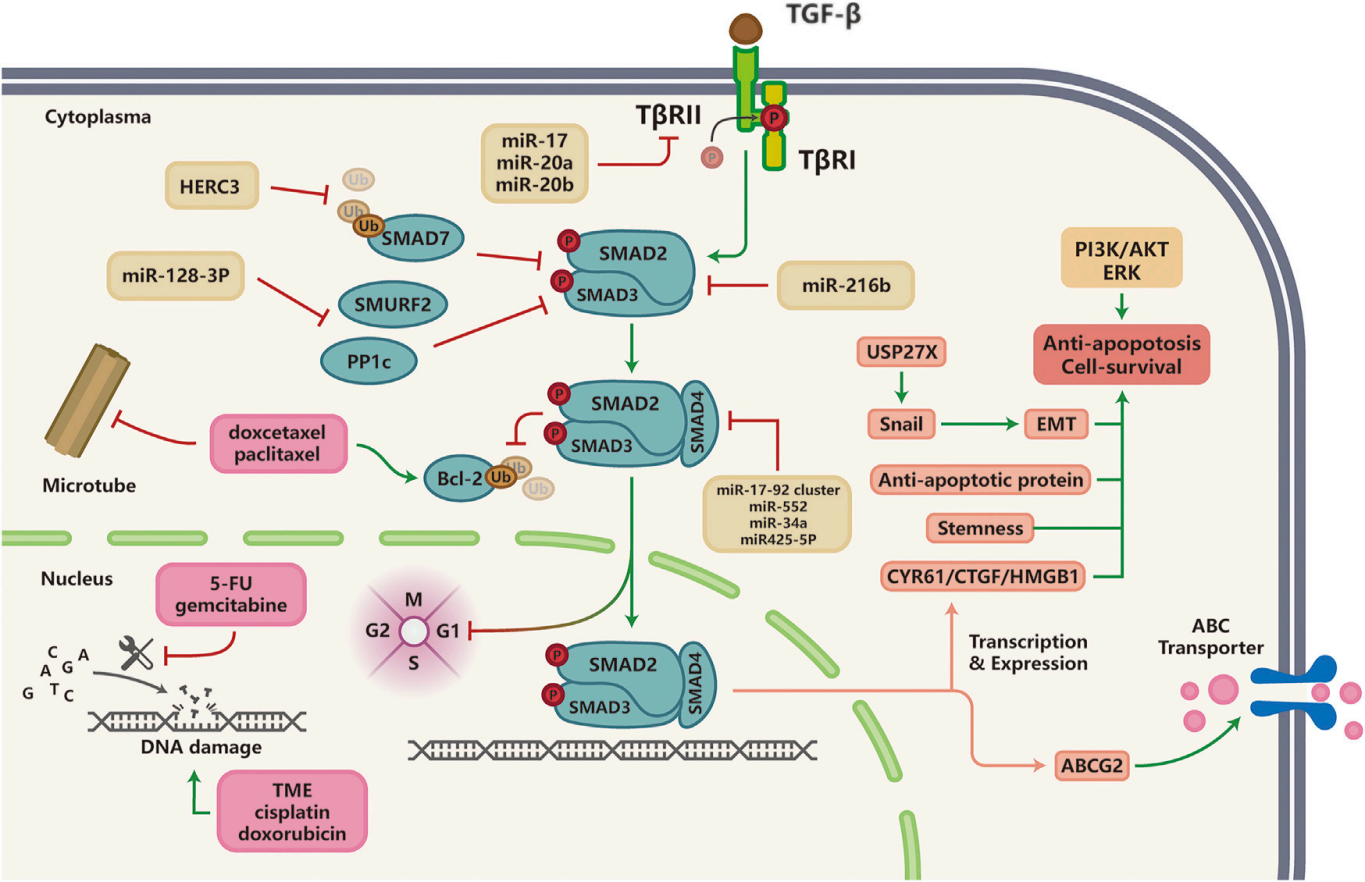
TGF-β signaling and resistance to chemotherapy; Multiple miRNAs are implicated in TGF-β-induced chemotherapy resistance in various cancer types by targeting components of the TGF-β pathway (SMAD2, SMAD3, SMAD4). Anti-microtubule drugs promote Bcl-2 protein ubiquitination, which could be inhibited by TGF-β signaling to induce taxane resistance in malignancies. Hyperactivation of TGF-β signaling pathway induces resistance to DNA damaging agents and anti-metabolites through the activation of alternative survival pathways or anti-apoptotic signaling such as PI3K/AKT and ERK pathways, as well as elevated expression of ABC multi-drug transporters to facilitate cancer cell survival and drug efflux, respectively.

The goal of chemotherapy is to eliminate highly proliferative cells but are non-specific compared with targeted therapy. Chemotherapy can be further divided into a few sub-categories based on their molecular mechanisms, including DNA damaging agents, anti-metabolites, and anti-microtubule agents. Emerging literature suggests that TGF-β signaling contributes to chemotherapy resistance in a variety of solid tumors. Here, we will summarize studies that reveal how TGF-β signaling induces chemotherapy resistance (Figure 3).

### DNA Damaging Agents

DNA damaging agents, such as cisplatin, temozolomide (TMZ), oxaliplatin (OXA), doxorubicin, and etoposide, can cause cell cycle arrest and apoptosis through directly alkylating DNA, inhibiting topoisomerases and impairing DNA repair. However, like many other types of cancer treatments, chemotherapy efficacy is often compromised by the development of drug resistance. Drug resistance can arise from mutations, epigenetic changes, and other cellular and molecular mechanisms that are not yet fully elucidated (Chen et al., 2010; Ali et al., 2013; Cai et al., 2017; Li et al., 2019b; Li et al., 2019c; Lambies et al., 2019; Taniguchi et al., 2020; Vu et al., 2020). Because of the important roles of TGF-β signaling in acquired resistance against DNA damaging agents in cancer patients, the mechanisms underlying these processes are of high interest as they can direct novel drug development.

Accumulating evidence suggests that resistance to DNA damaging agents is often associated with activation of TGF-β signaling through various mechanisms, particularly by miRNA-mediated regulation of TGF-β signaling (Cai et al., 2017; Sun et al., 2017; Chuang et al., 2019; Zhu et al., 2019; Chen et al., 2020; Huang et al., 2020; Vu et al., 2020). miR-128-3p, which was markedly up-regulated in cisplatin-resistant NSCLC cell lines, induced mesenchymal and stem-like properties by inhibiting two negative regulators of the TGF-β pathway, SMAD-specific E3 ubiquitin protein ligase 2 (SMURF2) and protein phosphatase 1c (PP1c), which resulted in the activation of TGF-β pathway, eventually leading to EMT and the development of cisplatin resistance (Cai et al., 2017). In colorectal cancer, miR-34a directly targets the 3′-UTR of SMAD4 and represses signaling via TGF-β/SMAD4. In OXA-resistant CRC patients, miR-34a is downregulated to enhance macroautophagy by activating the TGF-β/SMAD pathway (Sun et al., 2017). Another example of miRNA-mediated regulation of TGF-β signaling came from cigarette smoke condensate treated lung cancer cell lines, where miR-216b overexpression increased resistance to platinum-based therapy by downregulating SMAD3 to further restrain TGF-β-induced tumor suppression, as well as by overexpressing Bcl-2 to escape from apoptosis (Vu et al., 2020). Besides, other researchers found that the miR17 family (miR-17, miR20a, miR20b) mediated up-regulation of TGF-β/SMAD signaling pathway to confer cisplatin resistance in NSCLC (Jiang et al., 2014).

It is well known that TGF-β plays an essential role in EMT; therefore, people started to investigate if there was a link between EMT and acquired drug resistance in cancer. Recent studies demonstrated TGF-β regulated EMT and autophagy in chemotherapy-resistant cells (Fischer et al., 2015; Zheng et al., 2015; Jiang et al., 2016; Li et al., 2019c; He et al., 2019; Jiang et al., 2019; Ungefroren, 2019; Feng et al., 2020; Chen et al., 2021). Analysis of The Cancer Genome Atlas database (TCGA) and clinical data showed that in TMZ and X-ray treated-glioblastoma, the expression of HERC3 (the E3 ubiquitin ligase) was significantly up-regulated by autophagy inducers to promote degradation of SMAD7, thereby activating the TGF-β/SMAD signaling to promote EMT, cell survival, migration and chemoradio-resistance (Li et al., 2019c). In addition to promoting EMT, TGF-β also regulates the expression of autophagy-associated genes. For instance, TGF-β signaling was up-regulated in leptin-treated mesenchymal stem cells (MSC) to enhance the expression of autophagy-associated genes, which promoted cisplatin-resistance in OS cells (Feng et al., 2020). Similarly, in breast and pancreatic cancer cell lines, TGF-β signaling during EMT contributes to cisplatin resistance by up-regulating the expression of USP27X, which increases Snail1 protein stability (Lambies et al., 2019). *In vitro*, sustained TGF-β treatment induced cathepsin B (CTSB)-mediated degradation of Disabled-2 (Dab2), which activated autophagy and inhibited apoptosis by destabilizing the pro-apoptotic Bim protein, thereby modulating doxorubicin-resistance and tumor metastasis (Jiang et al., 2016). Interestingly, recent studies have unveiled that TGF-β signaling plays an important role in CSCs to mediate chemoresistance. Using an *in vitro* reporter system for lineage tracing, Oshimori and his colleagues (Oshimori et al., 2015) showed that very few TGF-β-responding squamous cell carcinoma stem cells (SCC-SCs) were sensitive to cisplatin treatment, suggesting that TGF-β signaling pathway mediated primary resistance in CSCs. In cisplatin-resistant oral squamous cell carcinoma (OSCC), TGF-β regulated cancer cell stemness through a SMAD-independent pathway: TGF-β inhibited the function of the tumor suppressor FOXO3a through the AKT pathway, which resulted in increased expression of stemness markers, such as SOX2 and ABCG2 (Li et al., 2019d); the same phenomenon was also observed in epirubicin-resistant three negative breast cancer (TNBC) cells (Xu et al., 2018).

### Anti-Metabolites and Anti-Microtubule Drugs

Fluorouracil (5-FU) and gemcitabine, two anti-cancer agents belonging to the anti-metabolite category, are widely used to obstruct critical metabolic pathways that are necessary for cancer cell proliferation and survival. Studies showed that TGF-β signaling was involved in resistance to anti-metabolite drugs. Similar to what was observed in cases of chemo-resistance against DNA damaging agents, miRNAs are involved in the activation of TGF-β signaling in cells treated with anti-metabolites and anti-microtubule drugs. Examples of miRNA regulators of TGF-β signaling include miR-423-5p, miR-552, and miR-17–92 cluster (miR-17, miR-18a, miR-19a, miR-19b, miR-20a and miR-92a) in prostate cancer, colorectal cancer, and pancreatic cancer (Cioffi et al., 2015; Zhao et al., 2019; Shan et al., 2020). Intriguingly, TGF-β/SMAD signaling activation directly induced G1 cell-cycle arrest in SCC cells, leading to the entry of tumor-propagating cancer cells (TPCs) into quiescence, which protected cancer cells from DNA damage caused by 5-FU treatment by adopting a condensed heterochromatic state (Brown et al., 2017). Activation of TGF-β signaling also indirectly promotes gemcitabine resistance through reduced expression of nucleoside transporters hENT1 and hCNT3, which are two critical genes that promote cellular uptake of drugs (Hesler et al., 2016). Moreover, hypoxia-inducible factor (HIF-1α) and cancer-associated fibroblasts (CAFs)-secreted TGF-β2 converge to activate the expression of hedgehog transcription factor GLI2 in CRC-CSCs, resulting in increased stemness/dedifferentiation and resistance to 5-FU (Tang et al., 2018). In addition, TGF-β induces the expression of cysteine-rich 61 (CYR61), connective tissue growth factor (CTGF) and high-mobility group box-1 (HMGB1), which facilitates chemotherapy resistance in cancers by promoting the expression of anti-apoptotic proteins (Hesler et al., 2016; Xian et al., 2017; Zhuang et al., 2017).

Microtubules are important components of almost all eukaryotic cells. Drugs such as colchicine, nocodazole, and taxane can cause cell cycle arrest by directly affecting the assembly and disassembly of microtubules in cells. Taxanes including docetaxel and paclitaxel are extensively used in the treatment of various solid tumors to disrupt microtubule function in tumor cells (Li et al., 2020a). Similar to the mechanism of resistance to anti-metabolic drugs, resistance to taxanes is associated with dysregulation of the TGF-β signaling pathway. For example, aurora-A kinase (AURKA) is overexpressed in TNBC to mediate TGF-β-induced EMT in docetaxel-resistant and paclitaxel-resistant breast cancer cells (Jalalirad et al., 2021). In breast cancer and ovarian cancer, TGF-β/SMAD signaling up-regulates the expression of obg-like ATPase 1 (OLA1) and ST3GAL1 (a sialyltransferase), leading to accelerated EMT, enhanced cancer stem-like features, and the expression of anti-apoptotic proteins such as cleaved caspase 3, Bcl2-associated protein X (Bax) and Bcl-2 (Wu et al., 2018; Jalalirad et al., 2021). Moreover, it was reported that bone-borne TGF-β induced acetylation of human Krüppel-like factor 5 (KLF5) by activating CXCR4, which resulted in osteoclastogenesis, bone metastases, and the development of docetaxel resistance, on the other hand, the inhibition of TGF-β and CXCR4 signaling promoted cell cycle arrest and apoptosis in advanced prostate cancer cells (Zhang et al., 2021).

## TGF-β Signaling and Resistance to Immunotherapy

**FIGURE 4:**
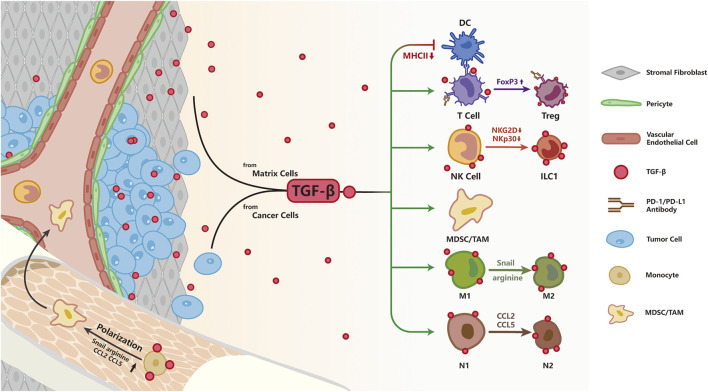
TGF-β signaling and resistance to immunotherapy; As an immunosuppression cytokine, TGF-β is secreted by both tumor and stromal cells. TGF-β signaling pathway directly inhibits T cell function by up-regulating the expression of FoxP3, converting cytotoxic T cells to Treg cells to restrain immune response. Besides, TGF-β impairs NK function by down-regulation of NKG2D and NKp30, two surface receptors directing NK cells to eliminate abnormal cells. TGF-β impairs antigen presentation in DC cells by decreasing MHCII expression. TGF-β signaling pathway polarizes macrophages to the pro-tumorigenic M2 phenotype by increasing Snail, converts N1 neutrophils to an N2 phenotype by up-regulation of arginine, CCL2, CCL5, and facilitates expansion of MDSCs leading to enhanced immune tolerance.

The immune system has developed a precise mechanism to recognize and purge malignant cells. However, in response to immune surveillance, some tumor cells evolve to escape the attack from the immune system by changing or decreasing the expression of tumor-specific antigens, up-regulating immune checkpoint proteins, and altering the expression of certain cytokines to facilitate immune evasion (Kennedy and Salama, 2020). To date, clinically approved cancer immunotherapy includes immune-checkpoint inhibitors, which target immune checkpoints such as cytotoxic lymphocyte-associated protein 4 (CTLA-4) or programmed cell death protein 1 (PD-1) and its ligand programmed death-ligand 1 (PD-L1), as well as chimeric antigen receptor T cell (CAR-T) therapy. These strategies aim to alleviate the suppression of the immune system by tumor cells, thereby reactivating anti-tumor responses and preventing immune escape (van den Bulk et al., 2018). Although cancer immunotherapy has made impressive progress in the treatment of a number of solid tumors and hematologic malignancies (Tumeh et al., 2014; Cristescu et al., 2018; Rodig et al., 2018), challenges persist as only a subset of patients with solid tumors are able to benefit from immunotherapy, owing to multiple factors such as the development of therapy resistance and interference from the intricate tumor microenvironment (TME). TGF-β is one of the most critical regulators of the TME; it is secreted by not only tumor cells but also multiple types of stromal cells including CAFs, tumor-associated macrophages (TAM), blood endothelial cells, MSC, lymphatic epithelial cells, and pericytes (Turley et al., 2015; Ganesh and Massagué, 2018). Interestingly, accumulating evidence suggests that TGF-β has an adverse role in immunotherapy response (Ganesh and Massagué, 2018; Batlle and Massagué, 2019; Larson et al., 2020). Here, we will provide a synopsis of studies on how TGF-β signaling modulates cancer immunotherapy response and discuss potential strategies to overcome TGF-β-induced immunosuppression (Figure 4).

TGF-β has been shown to regulate cellular functions of immunocytes including macrophages, neutrophils, bone marrow derived suppressor cells (MDSC), natural kill (NK) cells, dendritic cells (DCs) and T cells, by abolishing their cytotoxic function (Batlle and Massagué, 2019; Larson et al., 2020). TGF-β suppresses cellular functions of a variety of innate immune cells including macrophages, neutrophils, MDSC and NK cells, acting as an immune-suppressor in the TME; hyperactivation of the TGF-β signaling pathway polarizes macrophages to the pro-tumorigenic M2 phenotype by increasing Snail expression (Draghiciu et al., 2015; Zhang et al., 2016). In addition, activated TGF-β signaling converts N1 neutrophils to the immunosuppressive, pro-tumorigenic N2 phenotype by up-regulating production of arginine, CC chemokine ligand 2 (CCL2), CCL5 (Fridlender et al., 2009), while promoting the expansion of MDSCs resulting in immune tolerance (Batlle and Massagué, 2019). In addition, TGF-β was shown to block NK cell function by silencing the expression of NKG2D and NKp30 (Castriconi et al., 2003). TGF-β secreted by tumor cells facilitates the escape of tumor cells from immune surveillance by directly driving the conversion of NK cells into innate lymphoid cells type 1 (ILC1), which lacks cytotoxic function, or by impairing NKG2D-mediated cytotoxicity (Cortez et al., 2017; Gao et al., 2017; Lazarova and Steinle, 2019). DCs are the cardinal antigen presenting cells and the messenger between innate and adaptive immunity. By suppressing the expression of major histocompatibility complex II (MHC-II), TGF-β inhibits the ability of DCs to present antigens *in vitro* (Nandan and Reiner, 1997; Piskurich et al., 1998).

Other than the inhibition of cytotoxic functions of innate immunity as described above, TGF-β can also antagonize the adaptive immunity; and increasing evidence suggests that TGF-β signaling suppresses anti-tumor immunity by blocking the differentiation and functions of T helper1 (T_H_1), T helper 2 (T_H_2) CD4^+^ T cells and cytotoxic CD8^+^ T cells, while promoting the differentiation, function and survival of CD4^+^CD25^+^ forkhead box P3 (FoxP3) regulatory T cells (Tregs) cells (Nakamura et al., 2001; Thomas and Massagué, 2005; Tone et al., 2008). In healthy tissues, Tregs are present at a low level and suppress the function of T cells to maintain immune homeostasis. In activated Treg cells, the transmembrane glycoprotein A repetitions predominant (GARP) Protein is highly expressed and directs latent TGF-β to link with integrin avβ8 on the cell membrane to release active TGF-β, which contributes towards an immunosuppressive TME (Bouchard et al., 2021). Specific inhibition of TGF-β1 in GARP-expressing Treg cells was able to overcome resistance to PD-1/PD-L1 blockade in cancer patients (de Streel et al., 2020). Furthermore, to inhibit the release of active TGF-β in the TME, neutralizing antibodies were devised to target GARP or integrin avβ8, effectively reversing the adverse effect of TGF-β on T cells (Rachidi et al., 2017; Seed et al., 2021). Researchers also demonstrated that TGF-β suppressed T_H_2-mediated cancer immunity. Blocking TGF-β signaling in CD4^+^ T cells but not CD8^+^ T cells restrained tumor growth by remodeling the TME and inducing tumor vasculature reorganization, leading to cancer cell death; this process was dependent on the T_H_2 cytokine interleukin-4 (IL-4), but not the T_H_1 cytokine interferon-γ (IFN-γ). In TβRII-deficient CD4^+^ T cells, IL-4 promoted T_H_2 cells gene expression program to induce T cell activation and T_H_2 cells differentiation (Li et al., 2020b; Liu et al., 2020). The level of CD8^+^ T cells in the tumor parenchyma is a crucial factor in immunotherapy efficacy; TGF-β signaling in the TME has been implicated in the suppression of T-cell infiltration into tumors to compromise the efficacy of anti-PD-L1 antibody (Ganesh and Massagué, 2018). Other studies reported that TGF-β1 induced high expression of PD-1 and PD-L1 in T cells and tumor cells, respectively, to impair the anti-tumor activities of T cells and facilitate cancer immune evasion (Park et al., 2016; David et al., 2017; Tang et al., 2020). In addition to acquired resistance by activating alternative pathways of immune evasion, the lack of response to immunotherapy can sometimes be attributed to the presence of primary resistance in the tumor immune landscape. While mechanisms underlying primary resistance to cancer immunotherapy are yet to be fully characterized, inhibition of TGF-β signaling has been shown to overcome primary resistance to PD-1 blockade by altering the immunosuppressive TME (Martin et al., 2020; Siewe and Friedman, 2021).

To target TGF-β-mediated resistance to immunotherapy, several groups have tested the combination of TGF-β inhibitors with anti-PD-1/PD-L1 antibodies that were approved by the FDA for the treatment of multiple advanced cancers, including atezolizumab, durvalumab, and avelumab; results from these studies showed that combination treatment elicited higher anti-tumor activity in murine model and human cancer cell lines, such as breast cancer, colon cancer, SCC (Lan et al., 2018; Mariathasan et al., 2018; Tauriello et al., 2018; Dodagatta-Marri et al., 2019; Principe et al., 2019; Lind et al., 2020). Co-administration of TGF-β inhibitors and anti-PD-L1 antibody effectively reduced TGF-β signaling in stromal cells, resulting in improved T-cell penetration and more vigorous anti-tumor immunity to suppress urothelial tumor growth (Mariathasan et al., 2018). Similarly, the combinations of anti-CTLA4-TβRII or anti-PD-L1-TβRII elicited more pronounced anti-tumor responses than single treatments (Ravi et al., 2018). Upon expression of dominant-negative TβRII in CAR-T cells targeting prostate specific membrane antigen (PSMA), increased lymphocyte proliferation and exhaustion resistance were observed. This resulted in long-term *in vivo* persistence and enhanced infiltration of CAR-T cells into tumor sites, leading to improve tumor eradication in prostate cancer patient derived xenograft (PDX)-mouse model (Kloss et al., 2018). However, studies in mouse models of colon or pancreatic tumor demonstrated that combining anti-PD-1 and anti-TGF-β therapies improved long-term survival and delayed tumor growth in the MC38 murine colon carcinoma model, while failing to do so in the CT26 colon carcinoma model and KPC1 pancreatic tumor model (Sow et al., 2019; Bertrand et al., 2021). The above results suggest that special attention might be needed in selecting patients who would benefit the most from combination therapy.

### Combination Therapy: Opportunities and Challenges

Despite great improvements in the clinical application of chemotherapy, targeted therapy, as well as immunotherapy over the past few decades, the development of drug resistance has been proven inevitable. As aforementioned, multiple studies have suggested that TGF-β signaling was associated with enhanced drug resistance and tumor metastasis. As a result, researchers have started to explore the possibility of using TGF-β inhibitors in combination with other anti-cancer agents to treat patients with metastatic or recurrent tumors. So far, pre-clinical studies have demonstrated that combination therapy effectively blocked cancer cell proliferation and invasion *in vitro* (cell lines), *in vivo* (mouse models), and *ex vivo* (patient tumor explants). For example, combined regimens of sorafenib or erlotinib with TGF-β inhibitor effectively potentiated sorafenib by increasing HCC cells apoptosis and suppressed the motility of erlotinib-resistant NSCLC cells, respectively (Serizawa et al., 2013; Serova et al., 2015). TGF-β signaling was found to be activated in cells that survived paclitaxel treatment; and combining TGF-β pathway inhibitors with paclitaxel potently prevented recurrences of basal-like breast tumors *in vivo* (Bhola et al., 2013). Combining TGF-β inhibitor with immunotherapy has also shown promise in a number of pre-clinical studies (Mariathasan et al., 2018; Tauriello et al., 2018; Lind et al., 2020).

Although encouraging advances in treatment efficacy were observed in combining TGF-β pathway inhibitors with other anti-cancer agents in pre-clinical studies, successes in clinical trials remained rare and results were inconsistent to say the least. Treatment combinations involving a number of TGF-β inhibitors that were designed to bind to TGF-β receptors and inhibit receptor kinase activity, including AP12009, cilengitide, M200, LY2157299, NIS793, TEW-7197, have been tested in clinical trials (Table 1). Published results from these trials showed that the combination of LY2157299 with gemcitabine in metastatic pancreatic cancer (NCT01373164), as well as the combination of AP12009 with TMZ in Glioblastoma and anaplastic astrocytoma (NCT00431561) yielded encouraging outcomes. However, using cilengitide in combination with cisplatin and 5-FU to treat recurrent and/or metastatic hand and neck squamous cell carcinoma (HNSCC) resulted in no improvement in progression-free survival (PFS) and overall survival (OS) (NCT00705016) (NCT00689221). Furthermore, the efficacy of combining anti-TGF-β therapy with immunotherapy for the treatment of advanced solid tumor remains an unanswered question as data from clinical trials are not yet publicly available (NCT02423343) (NCT02947165).

**TABLE 1:** Overview of combination anti-TGF-β therapy with other cancer therapies in clinical trials.

Drug (target)	Clinical trial (Phase)	Status	Cancer type	Arms	Outcomes
AP 12009 (TGF-β2)	NCT00431561 (Phase II)	Completed	Glioblastoma and anaplastic astrocytoma	AP 12009 (10 mM) AP 12009 (80 mM) Temozolomide or procarbazine, lomustine, and vincristine	Improved PFS
					Improved OS (Results for responders regardless drug concentration administered)
Cilengitide also called EMD 121974 (integrins αvβ3 and αvβ5)	NCT00705016 (Phase III)	Completed	Head and Neck Squamous Cell Carcinoma	Cilengitide (2000 mg) cetuximab+5-FU + cisplatin	No improvement in PFS
				Cilengitide (2000 mg) cetuximab+5-FU + cisplatin	No improvement in OS
Cilengitide (integrins αvβ3 and αvβ5)	NCT00689221 (Phase III)	Completed	Glioblastoma	Cilengitide + temozolomide + radiotherapy	No improvement in PFS
				Temozolomide + radiotherapy	No improvement in OS
M200 (integrin α5β1)	NCT00635193 (Phases I/II)	Completed	Ovarian cancer and primary peritoneal cancer	Liposomal doxorubicin (40 mg/m^2^)	NA
				M200 (7.5 mg/kg)	
				Liposomal doxorubicin (40 mg/m^2^)	
				M200 (15.0 mg/kg)	
				Liposomal doxorubicin (40 mg/m^2^)	
LY2157299 also called galunisertib (TβRI)	NCT01220271 (Phases I/II)	Completed	Glioblastoma	Phase I	NA
				LY2157299 (160 mg) + radiotherapy + temozolamideLY2157299 (300 mg) + radiotherapy + temozolamide	
				Phase II	
				LY2157299 (established dose) radiotherapy + temozolamideRadiotherapy + temozolamide	
LY2157299 (TβRI)	NCT02154646 (Phase I)	Completed	pancreatic cancer	galunisertib + gemcitabine	NA
LY2157299 (TβRI)	NCT01373164 (Phases I/II)	Completed	pancreatic cancer	galunisertib + gemcitabine vs placebo + gemcitabine	Improved OS
LY2157299 (TβRI)	NCT02734160 (Phase I)	Completed	pancreatic cancer	galunisertib + durvalumab (PD-L1 antibody)	NA
LY2157299 (TβRI)	NCT02423343 (Phases I/II)	Completed	NSCLC and HCC	galunisertib + nivolumab (anti-PD-1)	NA
LY2157299 (TβRI)	NCT02178358 (Phases I/II)	Completed	HCC	monotherapy vs combination with sorafenib or placebo + sorafenib	NA
TEW-7197 (TβR1)	NCT03074006 (Phases I/II)	Completed	pancreatic cancer	combination with FOLFOX in pancreatic cancer patients	NA
NIS793 (TGF-β)	NCT02947165> (Phase I)	Completed	Breast Cancer	NIS793 + PDR001 (anti-PD-1)	NA
			Lung Cancer		
			Hepatocellular Cancer		
			Colorectal Cancer		
			Pancreatic Cancer		
			Renal Cancer		

5-FU, 5-fluoracil; PFS, progression-free survival; OS, overall survival; NA, not available (results are not publicly available).

A few factors might account for the suboptimal outcomes of anti-TGF-β therapies in a number of clinical trials. First of all, the animal models used in pre-clinical studies might not reflect the complexity of the disease in human patients; for instance, in models using patient-derived tumor xenografts, the TME in the mouse model can habour dramatic differences from the human TME, such that drugs might be effective in treating tumors in animal models but fail to do so in human patients. Second, TGF-β signaling is highly dynamic; feedback loops that regulate the activity of TGF-β signaling have been reported and oscillations in TGF-β signaling have been modeled and tested *in vitro* (Zi et al., 2011; Warmflash et al., 2012; Wegner et al., 2012). As a result, the effectiveness of antagonizing TGF-β signaling in an attempt to suppress cancer cell survival and drug resistance might be complicated by the innate fluctuations in TGF-β signaling. Furthermore, the heterogeneity in tumor cells can also contribute to heterogeneity in the response towards anti-TGF-β therapies. A study by Giampieri et al. demonstrated that single tumor cells activated TGF-β signaling locally and transiently, such that single cell motility, rather than collective movement, was enhanced (Giampieri et al., 2009). Importantly, inhibition of TGF-β signaling prevented single cell motility but not collective movement of tumor cells; cells expressing the dominant negative TβRII were incapable of metastasizing to the lung via blood vessels, while still being able to disseminate to lymph nodes via collective invasion (Giampieri et al., 2009). In addition, with TGF-β being a key regulator in the maintenance of tissue homeostasis, on-target cardiovascular toxic side effects and formation of benign tumors in response to the targeting of TGF-β signaling have been reported (Colak and Ten Dijke, 2017). Generally, although TGF-β inhibitors in combination with other anti-cancer treatments have yielded encouraging results in pre-clinical studies, thorough characterization of the mode of action and response to these inhibitors, as well as a better understanding of the pleitropic nature of TGF-β signaling are important to optimize the survival benefits from using TGF-β inhibitors and to facilitate the bench-to-bedside transition for anti-TGF-β therapy (Ciardiello et al., 2020).

## Conclusion

The aberrant activation of TGF-β signaling plays a complex role in tumor progression, especially in the development of resistance towards cancer therapy. TGF-β induces drug resistance in targeted and chemotherapy by activating alternative survival pathways or anti-apoptotic signaling. On the other hand, other than activating TGF-β signaling pathways to induce drug resistance as delineated above, under certain circumstances, down-regulation of TGF-β signaling pathway has also been associated with enhanced drug resistance (Faião-Flores et al., 2017; Bugide et al., 2020; Vu et al., 2020). For example, down-regulation of TGF-β signaling through the inhibition by MITF (Microphthalmia-associated transcription factor) can confer MEKi resistance in melanoma (Smith et al., 2013). Reduced levels of SMAD3 or loss of SMAD4 suppressed the function of TGF-β-induced expression of tumor suppressor genes, resulting in the expression of anti-apoptotic proteins Bcl2 and Bcl-W, and enhanced cancer cell survival to confer platinum-resistance in NSCLC and 5-FU resistance in CRC, respectively (Zhang et al., 2014; Vu et al., 2020). Furthermore, some researchers suggested that TGF-β could serve as an important immune checkpoint in subverting “hot tumors,” which had more infiltrating T-cells, into “cold tumors,” which had lower immune infiltrates (Larson et al., 2020).

Therapeutic strategies using TGF-β inhibitors are making a tardy progress because of the dichotomous functions of TGF-β signaling in cancer. One of the two main concerns is that inhibitors of TGF-β signaling may impede cancer progression in the later stages of cancer but fail to suppress tumors at early stages. Another concern is that in clinical trials, the application of TGF-β inhibitors may result in off-target toxicity, especially cardiac toxicity (Turley et al., 2015) and dose-limiting toxicities (NCT01646203). In conclusion, although TGF-β inhibitors in combination with cancer therapy especially immunotherapy have shown great promise, thorough characterization of these inhibitors, as well as careful stratification and selection of patients are still required for cancer patients to truly benefit from it.
